# Flexible wire-shaped strain sensor from cotton thread for human health and motion detection

**DOI:** 10.1038/srep45013

**Published:** 2017-03-21

**Authors:** Yuan-Qing Li, Pei Huang, Wei-Bin Zhu, Shao-Yun Fu, Ning Hu, Kin Liao

**Affiliations:** 1 College of Aerospace Engineering, Chongqing University, Chongqing 400044, P. R. China; 2 The State Key Laboratory of Mechanical Transmissions, Chongqing University, Chongqing 400044, P. R. China; 3 Department of Mechanical Engineering, Khalifa University of Science, Technology, & Research, Abu Dhabi 127788, UAE.

## Abstract

In this work, a wire-shaped flexible strain sensor was fabricated by encapsulating conductive carbon thread (CT) with polydimethylsiloxane (PDMS) elastomer. The key strain sensitive material, CT, was prepared by pyrolysing cotton thread in N_2_ atmosphere. The CT/PDMS composite wire shows a typical piezo-resistive behavior with high strain sensitivity. The gauge factors (GF) calculated at low strain of 0–4% and high strain of 8–10% are 8.7 and 18.5, respectively, which are much higher than that of the traditional metallic strain sensor (GF around 2). The wire-shaped CT/PDMS composite sensor shows excellent response to cyclic tensile loading within the strain range of 0–10%, the frequency range of 0.01–10 Hz, to up to 2000 cycles. The potential of the wire senor as wearable strain sensor is demonstrated by the finger motion and blood pulse monitoring. Featured by the low costs of cotton wire and PDMS resin, the simple structure and fabrication technique, as well as high performance with miniaturized size, the wire-shaped sensor based on CT/PDMS composite is believed to have a great potential for application in wearable electronics for human health and motion monitoring.

Smart devices for monitoring physiological and biomechanical signals of the human body are gaining increasing importance for personalized healthcare^1^,^2^, particularly wearable strain sensors, that can be used to monitor joint and muscle motion with the aim of sensing posture, movement, breathing, heart beat, and blood pulse^3^,^4^,^5^. Conventional strain sensors based on thin metal-wires and semiconductors are fragile and rigid, restricting their applications as wearable devices^6^. Although progress has been made in developing flexible strain sensors based on conductive elastomers, their bulky size prevents sweat and air from body to pass through them freely, thus they may not be wearable^7^,^8^,^9^,^10^,^11^.

The continued miniaturization of wearable electronics requires smaller, lighter, and more flexible strain sensors to meet the growing demands for personalized healthcare market^12^. Among various attempts, the development of wire-shaped strain sensors are increasingly appreciated as a reliable strategy to leap over the geometrical restrictions of traditional bulk devices^13^,^14^,^15^,^16^,^17^,^18^. The wire-like shape enables exceptional properties, including small dimension, light weight, and high flexibility with good wearability to be imparted on the strain sensors. Such unique architecture also renders great design versatility compared to conventional wearable devices, since they can be fabricated into various desired shapes and located at different places^19^,^20^. Currently, various conductive materials, such as intrinsic conductive polymer^21^, silver^20^ and gold^13^ nanoparticles, silver nanowires^14^, carbon nanotubes^15^, and graphene^16^,^17^,^18^ etc. have been exploited to fabricate the wire-shaped strain sensors. However, high cost of raw materials, complicated preparation procedures drastically hamper the scale-up production of these wire-shaped strain sensors^22^. Thus development of wire-shaped wearable strain sensor with low-cost and simple fabrication techniques remains a challenge.

Due to their environmental benignity, low-cost, good electrical conductivity, and large-scale production capability, carbon-based materials from biological materials have received extensive research attention^23^,^24^,^25^,^26^,^27^. Cotton, a widely used natural material with a composition of 90–95% cellulose is both low-cost and sustainable, making it a promising raw material to fabricate carbon based materials^28^,^29^. Recently, a simple yet highly sensitive wearable pressure sensor using cotton as raw materials was developed by our group. It shows a maximum sensitivity of 6.04 kPa^−1^ with a wide working pressure that ranges up to 700 kPa^22^. However, the typical size of the pressure sensor prepared is 15 × 50 mm^2^ with a thickness of 4 mm, thus downsizing is necessary to expand its applications in wearable devices.

In this work, we describe the fabrication of a wire-shaped strain sensor using cotton thread as raw material. The strain sensor composes of a flexible and piezo-resistive carbon thread/polydimethylsiloxane (CT/PDMS) composite wire connected with two copper wires as electrodes. The sensor’s simple structure combined with miniaturized size and high strain sensitivity make it highly promising as wearable sensor for human health and motion monitoring.

## Experimental section

### Preparation of carbon threads (CT) and CT/PDMS sensors

Regular cotton sewing threads (Ji-Meng-Se, Rizhao, China) in two plies with a linear density of 1160 Tex (g/km) were used to prepare the CTs. First, the cotton threads were carbonized at 800 °C for 1 h in N_2_ atmosphere, and the CTs obtained were cut into 40 mm long pieces. Then two monofilament copper wires with a diameter of 0.24 mm as electrodes were soldered with silver paste (PELCO, TED PELLA) at the two ends of a piece of CT sample. Finally, the CT-with-electrodes assembly were encapsulated with polydimethylsiloxane (PDMS) elastomer by dip coating. PDMS resin (Sylgard 170, Dow Corning) was prepared by mixing PDMS base and catalyst in the mass ratio of 1:1. To cure the PDMS resin, the CT-with-electrodes coated with PDMS resin were heated at 80 °C for 30 min.

### Characterizations

The morphologies of CTs and CT/PDMS composite wires were examined by a scanning electron microscope (SEM, JEOL7610F). Electrical conductivities of the CTs and the CT/PDMS wires were measured with a two-probe method using a digital multimeter (ADM-930, 0.1 Ω ~ 40 MΩ). The tensile behavior of the CT/PDMS wire was investigated using a Microforce Tester (Instron 5948) at a loading rate of 1 mm/min. To study the strain-sensing performance, the wire-shaped sensor based on CT/PDMS wire was fixed between the two grips of the Microforce Tester, while each electrode of the sensor was connected with one of the electrodes of an electrochemical workstation (Autolab 302 N). When tensile strain was applied to the wire-shaped sensor, the change in current from the sensor was recorded by the electrochemical workstation. The working voltage of the sensor was set at 1 V. The relative change of the resistance (RCR, %) is calculated based on the resistance measured: ∆R/R_0_ = (R_s_ − R_0_)/R_0_, where R_0_ and R_s_ are the resistance without and with applied strain, respectively. Furthermore, the gauge factors (GF) of the strain sensor, defined as δ(ΔR/R_0_)/δS, where *S* denotes the applied strain, are calculated based on the RCR-strain curves plotted.

## Results and Discussion

**Figure 1:**
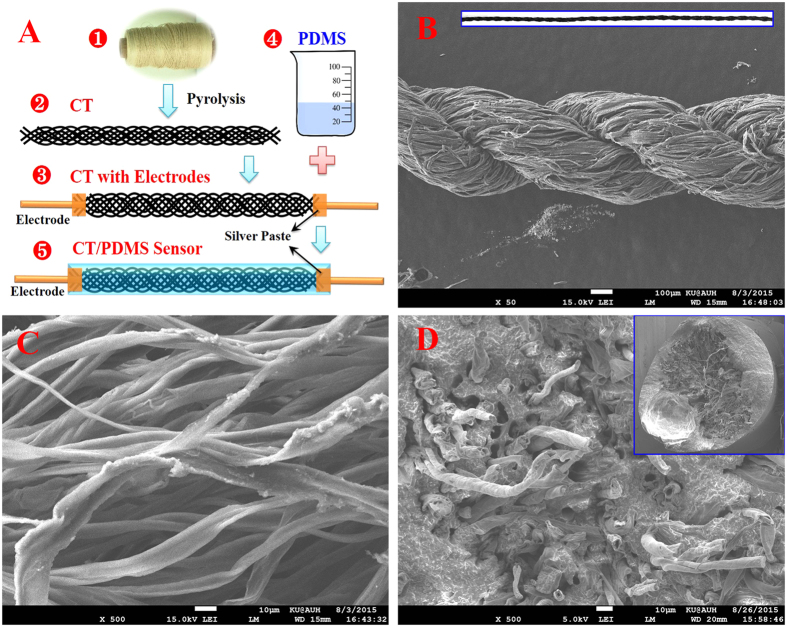
(**A**) Schematics of the fabrication of the wire-shaped strain sensor: (1) cotton thread, (2) CT, (3) CT with electrodes, (4) PDMS resin and (5) CT/PDMS sensor. SEM images of CTs with low (**B**) and high (**C**) magnification, and the fracture surface of CT/PDMS composite wire (**D**).

The use of cotton threads by humans in fabric and textile can be traced back five millennia^30^. Regular cotton threads are formed with multiple cellulose microfibers bundled together^31^, which can be converted into carbon via a simple pyrolysis process. As indicated in Fig. 1(A), CTs are prepared by directly heating the cotton thread at 800 °C for 1 h in N_2_ atmosphere. The shape of CTs obtained after pyrolysis is identical with that of starting cotton threads, but its post-pyrolysis volume is only around 50% of the starting cotton thread due to the evaporation of volatile organic species^22^. As shown in Fig. 1(B), the CTs prepared have a two ply structure, composed of multiple carbon fibers derived from cotton fibers. High magnification SEM images, as shown in Fig. 1(C), reveal that those fibers, with a diameter around 10 μm, are loosely twisted and ample of free space is observed between the microfiber bundles. Moreover, the CTs exhibit an average electrical conductivity of 1.24 S/m, which makes it an alternative candidate as the strain resistive material. In order to fabricate the flexible wire-shaped sensor, the CTs with copper electrodes were encapsulated by PDMS resin with a dip coating method. The inner structure of the CT/PDMS composite wire prepared is shown in Fig. 1(D). The outer layer of the CT is fully covered by PDMS, and the free space inside CT is also soaked with PDMS resin, which endows the brittle CT with flexibility, environmental and mechanical stability.

**Figure 2:**
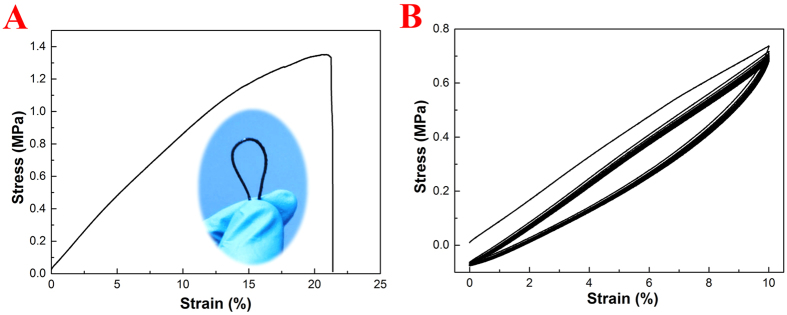
(**A**) Typical tensile stress-strain curve of the CT/PDMS composite wire, inset shows the flexibility of the composite wire. (**B**) The stress-strain curve of the CT/PDMS composite wire under cyclic tensile straining.

As shown in the inset of Fig. 2(A), the CT/PDMS composite wire is highly flexible, it can endure repeated bending without any visible damage. Tensile tests were performed to evaluate the mechanical behaviour of the CT/PDMS composite wire, and the typical strain-stress curve is presented in Fig. 2(A). The average tensile strength, Young’s modulus, and elongation-at-break of the CT/PDMS composite wire is 1.21 MPa, 15.8 MPa, and 16.7%, respectively. The relatively low Young’s modulus indicates that the straining of the CT/PDMS is sensitive to stress, which is an advantage for the fabrication of highly sensitive strain sensor. The CT/PDMS composite wire is not only flexible, but also repeatedly stretchable. Figure 2(B) shows the behavior of a CT/PDMS composite fiber under 10 loading-unloading stretching cycles. After the first loading-unloading cycle, the rest of the hysteresis are almost the same, but the stress of the CT/PDMS composite wire does not return to zero and maintains a constant offset in the rest of the cycles, indicating that internal structural rearrangement may have occurred during the first cycle and then it tends to be stabilized afterwards as the hysteresis does not show much deviation from each other.

**Figure 3:**
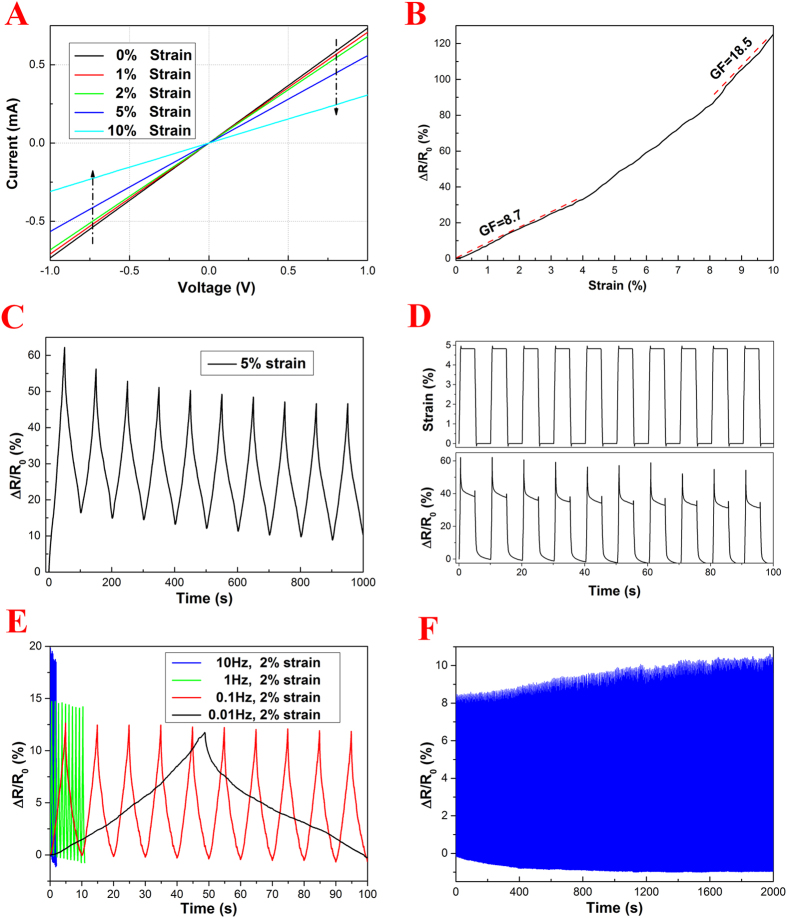
(**A**) Current-voltage curves of the CT/PDMS composite wire under various static strain. RCR response of the CT/PDMS composite wire with single tensile loading (**B**), tensile loading-unloading cycles (**C**), loading-hold-unloading-hold cycles (**D**). (**E**) Effect of frequency on the RCR response of the CT/PDMS composite wire. (**F**) Reliability test of the CT/PDMS composite wire with 2% peak strain and 1 Hz frequency, up to 2000 cycles.

Although the PDMS surface layer itself is nonconductive, as a whole, the CT/PDMS composite wire shows an electrical conductivity of 0.68 S/m, indicating that the conductive paths of the CT are well preserved after the infusion of PDMS resin. More importantly, the CT/PDMS composite wire exhibits a piezo-resistive behavior, which was further investigated by an electrochemical workstation coupled with a microforce tester. As shown in Fig. 3(A), the current-voltage (I-V) curves of the CT/PDMS composite wire at constant strain are linear in the voltage range of −1 to 1 V, indicating that the resistance of CT/PDMS composite is constant with an ohmic behavior. Meanwhile, the slope of the I-V curves decreases with an increase of applied strain, from 0 to 10%, indicating a rise in resistance, which confirms the piezo-resistive behavior of the CT/PDMS composite wire. The RCR response of the CT/PDMS composite wire to tensile loading is presented in Fig. 3(B). It is seen that the RCR of the CT/PDMS monotonically increases with an increase of the strain in the range of 0–10%. Within the strain range of 0–4%, the RCR of CT/PDMS composite wire exhibits a fairly linear relation with the applied strain (indicated by the red dotted line in Fig. 3(B)), and a GF of 8.7 is calculated. The slope of the RCR-strain curve at higher strains is obviously larger than that at lower strains, and the GF calculated within the strain range of 8–10% is 18.5. These results indicate that the sensitivity of the CT/PDMS composite wire fabricated is 4 (at 0–4% strain) and 9 (8–10% strain) times more sensitive than conventional metallic strain gauge (GF around 2).

To fully unveil the performance of the CT/PDMS composite wire as a strain sensor, its RCR response to tensile loading-unloading cycles was investigated. As shown in Fig. 3(C), the CT/PDMS composite wire is highly sensitive to the cyclic strain applied. At the first cycle, a RCR peak value of 62% is achieved at a strain of 5%, and a RCR offset of 16% remains after returning the strain to 0%. Then a slight drop in both the peak and offset of the RCR is seen in the next few cycles, due possibly, to stress relaxation. The RCR-time curve seems to be stabilized after a few cycles, which is synchronized with the response of stress under cyclic strain. The rise in electrical resistance with applied strain maybe interpreted as follows. With applied tensile loading, discontinuities in the conductive pathways start to appear within the CT/PDMS composite wire, and the amount of discontinuities increases with an increase of applied load, which results in the surge of electrical resistance. After releasing the wire to its strain-free condition, most of the disconnected conductive pathways recover to their initial states. However, the some broken carbon fibers result in a permanent contact disruption, which manifested as the increase of overall electrical resistance indicated by the RCR offset. After a few cycles, the internal structure of the CT/PDMS is stabilized as discontinuities in conductive pathways cease to form any more. Furthermore, while the responses of the CT/PDMS composite wire under different applied strain from 1% to 10% are quite similar, the RCR peak value has increased from 5% to 132% (Figs 3(B), S1).

The behavior of the CT/PDMS composite wire under loading-hold-unloading-hold cycles was also studied. As shown in Fig. 3(D), the RCR response curve synchronizes with the applied strain curve. However, within the brief strain holding period, the RCR of the composite wire exhibits a slow recovery due to stress relaxation. It is known that the relaxation time of PDMS elastomer is in the scale of 10 s, within the strain holding period, the electrical conductive path discontinuities in the CT/PDMS are reconnected along with the relaxation of the internal stress.

The effect of loading frequency on the RCR response of the CT/PDMS wire is shown in Fig. 3(E). Within the frequency range of 0.01–10 Hz, the shape of the RCR response curves agrees well with that of the strain curve applied. However, the amplitude of the RCR at high frequency is obviously higher than that at low frequency. For instance, the peak RCR values under 0.01 Hz and 10 Hz are 12% and 19%, respectively. The frequency dependence of the RCR amplitude is typical of a flexible piezo-resistive sensor based on PDMS matrix. It is known that the internal stress generated for polymeric materials at high strain rate is essentially larger than that at low strain rate because of chain entanglement. With a constant maximum strain of 2%, the strain rate applied at 10 Hz is 1000 times that of 0.01 Hz, which results in a significant stress difference and the corresponding RCR amplitude difference.

To evaluate the durability of the CT/PDMS composite wire as a sensor, 2000 tensile cycles with a maximum tensile strain of 2% at 1 Hz were performed. Within the 2000 cycles studied, the shape of RCR curve agreed well with the profile of the applied strain, indicating the sensor’s excellent reliability. At the same time, as shown in Fig. 3(F), the amplitude of RCR increases from 8.5% to 12% within the first 1500 cycles, and it stabilizes afterwards. Moreover, the CT/PDMS wire not only shows high sensitivity to tensile strain (Figure S2), it is also sensitive to cyclic compressive and bending strain.

**Figure 4:**
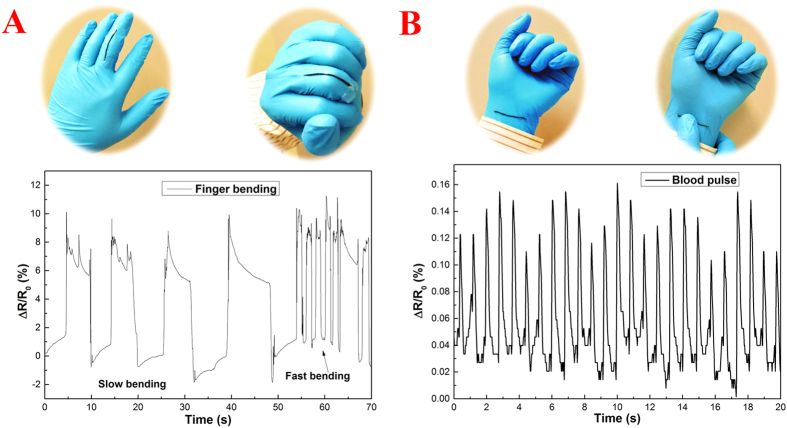
Application of wire-shaped sensor in finger motion (**A**) and blood pulse (**B**) monitoring.

In order to demonstrate the potential applications of the wire-shaped sensor based on CT/PDMS composite wire, the finger motion and blood pulse of a human adult were monitored by simply attaching the wire sensor on the finger and wrist of a testee, respectively. As shown in Fig. 4(A), when fully flexing the finger, a maximum RCR change around 10% is observed. Additional information, such as flexing-stretching speed and the holding time under stretching or flexing, can also be analyzed from the RCR data tracked, which will be useful in human-machine interaction with motion control. Moreover, the blood pulse of the adult testee within 20 s is monitored (Fig. 4(B)). Although the blood pulse is very subtle with a maximum RCR peak value only around 0.15%, the RCR output signal exhibits high reliability and good reproducibility. An average blood pulse of 78 beats per minute is calculated based on the collected data, which agrees well with the typical heartbeat of a healthy adult.

## Conclusions

In this work, the fabrication and application in human motion and health detection of a wire-shaped strain sensor based on the CT/PDMS composite are demonstrated. The miniaturization of the strain sensor is realized with the design of conductive CT originated from cotton thread as strain sensitive material. Encapsulation of with PDMS gives the resultant CT/PDMS composite wire of both high flexibility and excellent piezo-resistivity. The gauge factor of the CT/PDMS composite wire is much higher than that of traditional metallic strain sensors. The wire-shaped sensor based on CT/PDMS is highly sensitive to cyclic tensile loading applied within the frequency range of 0.01 to 10 Hz up to 2000 cycles. Furthermore, the demonstration of the wire-shaped senor as wearable strain sensor is performed by finger motion and blood pulse detection. Due to its excellent flexibility and high stretchability, the wire-shaped sensor can be integrated into textile, which may impart smart functionality, such as sensing and monitoring, to garments. Further studies are necessary to explore the full potentials of CT/PDMS wire sensor in human health and motion detection.

## Additional Information

**How to cite this article**: Li, Y.-Q. *et al*. Flexible wire-shaped strain sensor from cotton thread for human health and motion detection. *Sci. Rep.* **7**, 45013; doi: 10.1038/srep45013 (2017).

**Publisher's note:** Springer Nature remains neutral with regard to jurisdictional claims in published maps and institutional affiliations.

## Supplementary Material

Supplementary Information
