# Supplementary material for: Flexible wire-shaped strain sensor from cotton thread for human health and motion detection
Source: Sci Rep. 2017 Mar 21;7:45013. doi: 10.1038/srep45013 (PMC5359581; doi:10.1038/srep45013)
Supplement: Supplementary Information [file srep45013-s1.pdf]

Flexible wire-shaped strain sensor from cotton thread for human health and motion detection

Yuan-Qing Li[1], *Pei Huang*[1], *Wei-Bin Zhu*[1], *Shao-Yun Fu*[1*], *Ning Hu*[1,2], *and Kin Liao*[3*]

[1]College of Aerospace Engineering, Chongqing University, Chongqing 400044, P. R. China.

[2]The State Key Laboratory of Mechanical Transmissions, Chongqing University, Chongqing 400044, P. R. China

[3]Department of Mechanical Engineering, Khalifa University of Science, Technology, & Research, Abu Dhabi 127788, United Arab Emirates.

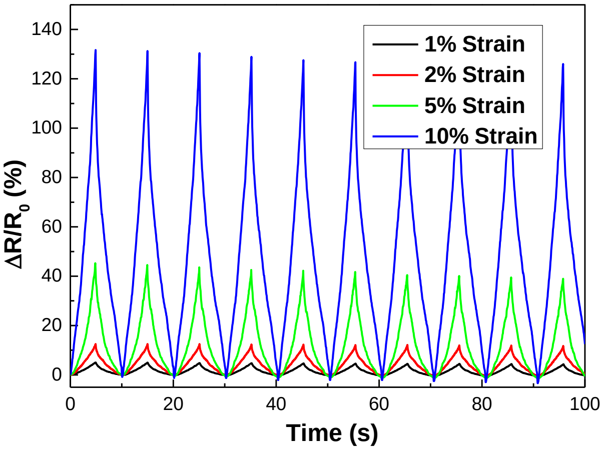

Figure S1. RCR response of the CT/PDMS composite wire to different cyclic strain.

Correspondence and requests for materials should be address to S.-Y.F. (syfu@cqu.edu.cn) or K.L. (kin.liao@kustar.ac.ae)

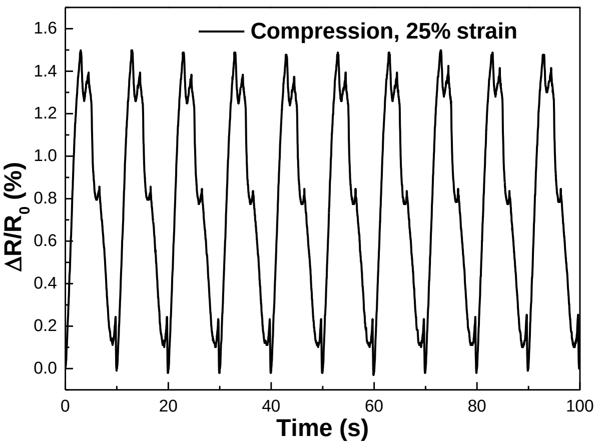

(A)

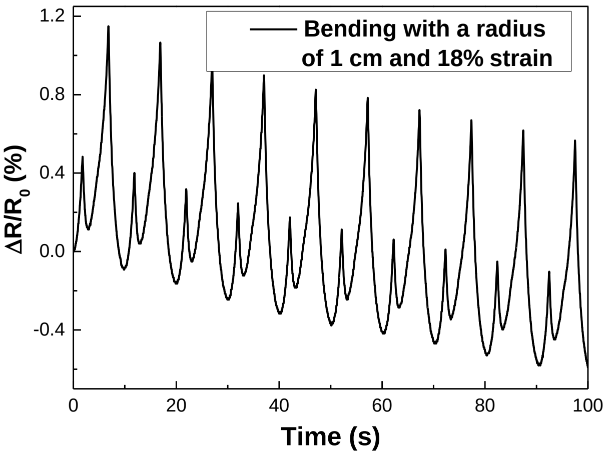

(B)

Figure S2. RCR response of the CT/PDMS composite wire to cyclic compressive (A) and bending (B) strain.
